# Designing Novel Compounds for the Treatment and Management of RET-Positive Non-Small Cell Lung Cancer—Fragment Based Drug Design Strategy

**DOI:** 10.3390/molecules27051590

**Published:** 2022-02-28

**Authors:** Priyanka Ramesh, Shanthi Veerappapillai

**Affiliations:** Department of Biotechnology, School of Bio Sciences and Technology, Vellore Institute of Technology, Vellore 632014, India; priyanka.r@vit.ac.in

**Keywords:** RET protein, docking, RF-Score, molecular dynamics, DFT calculations

## Abstract

Rearranged during transfection (RET) is an oncogenic driver receptor that is overexpressed in several cancer types, including non-small cell lung cancer. To date, only multiple kinase inhibitors are widely used to treat RET-positive cancer patients. These inhibitors exhibit high toxicity, less efficacy, and specificity against RET. The development of drug-resistant mutations in RET protein further deteriorates this situation. Hence, in the present study, we aimed to design novel drug-like compounds using a fragment-based drug designing strategy to overcome these issues. About 18 known inhibitors from diverse chemical classes were fragmented and bred to form novel compounds against RET proteins. The inhibitory activity of the resultant 115 hybrid molecules was evaluated using molecular docking and RF-Score analysis. The binding free energy and chemical reactivity of the compounds were computed using MM-GBSA and density functional theory analysis, respectively. The results from our study revealed that the developed hybrid molecules except for LF21 and LF27 showed higher reactivity and stability than Pralsetinib. Ultimately, the process resulted in three hybrid molecules namely LF1, LF2, and LF88 having potent inhibitory activity against RET proteins. The scrutinized molecules were then subjected to molecular dynamics simulation for 200 ns and MM-PBSA analysis to eliminate a false positive design. The results from our analysis hypothesized that the designed compounds exhibited significant inhibitory activity against multiple RET variants. Thus, these could be considered as potential leads for further experimental studies.

## 1. Introduction

Cancer is a worldwide life-threatening concern that is characterized by uncontrolled and progressive cellular divisions. It is the largest cause of mortality in the world, with 15 million new cases and 8.2 million deaths per year [1]. Among them, lung cancer is the most frequently occurring cancer with a high mortality rate of about 21% in men and 15% in women. The incidence of lung cancer is highly prevalent in Eastern and Western Asia, parts of Europe, and Northern America. Despite the advancements in primary lung cancer treatment technology, it still accounts for 1.3 million deaths globally in 2020 [2].

Rearranged during transfection (RET), an independent oncogenic driver belonging to the tyrosine kinase receptor family, is responsible for renal morphogenesis, the maintenance of spermatogonial stem cells, and the development of neuroendocrine neural tissues. The RET receptor consists of an intercellular domain, a transmembrane, and an extracellular region. The activation of RET protein involves the formation of ternary complexes with glial cell line-derived neurotrophic factors and its co-receptors, autophosphorylation of tyrosine kinase domain of RET, and finally resulting in the activation of downstream pathways that are implied in cell growth, differentiation, and proliferation [3]. Recently, genetic alterations of RET have been identified in diverse cancer types [4]. Among the specified types, medullary thyroid cancer, and non-small cell lung cancer (NSCLC) patients have been reported to contain a high frequency of RET mutations.

Furthermore, the gatekeeper mutation (V804M and V804L), solvent front (G810A, G810R, H810S and G810C), and other mutations including S904F, Y806C, Y806N, and V738A were observed to be the key factors for disease burden in NSCLC [5]. Adding together, RET fusion is observed in 1–2% of young non-smoking NSCLC individuals with a high risk of metastasis in the brain. For instance, the RET protein was found to be overexpressed on fusion with other kinases such as coiled-coil domain-containing-6 (CCDC6) and kinesin family 5B (KIF5B) [6].

Several multiple kinase inhibitors (vandetanib, cabozantinib, RXDX-105, and levantinib) and RET-specific inhibitors (pralsetinib and selpercatinib) with modest inhibitory activity have been developed for the treatment of RET-positive lung cancer patients. In spite of the dose-limiting off-target effect and response durability, the solvent front and gatekeeper mutations cause resistance to the majority of existing inhibitors [7,8,9,10]. In addition, Drusbosky et al. has recently reported that immunotherapy has a limited effect on patients with RET fusions [6]. Hence, developing novel drug candidates as RET inhibitors is crucial for better patient care.

Currently, drug development relies mainly on large-scale screening of chemical libraries against various biological targets. Among them, high-throughput screening (HTS) strategies have been found to have significant advancements [11]. For instance, Parate et al. and her research group investigated natural compounds using pharmacophore-based virtual screening approach to identify potent RET inhibitors for cancer therapeutics [12]. Similarly, a 3D-QSAR study was carried out by Pathak et al. and his team to establish the affinity of 1,3,5-triazine derivatives as cancer inhibitors against RET receptors [13]. Although HTS have successful applications in drug discovery, it has some limitations including a low hit rate and limited coverage of chemical space [11]. Hence, computational fragment-based drug designing (FBDD) has become a powerful and important technique for optimizing and discovering novel drug leads.

The complete exploration of the protein’s active site in this strategy results in an easy binding of fragments than the large molecules that were identified through the HTS process. Moreover, the fragment-based libraries achieve a higher success rate than the conventional HTS method, owing to its higher binding affinity towards the protein [14]. Thus, a fragment-based drug design strategy has been popular in recent years to develop novel and potent inhibitors against the target proteins [15,16]. For instance, a FBDD study by Ahmad and his research group proposed three hit molecules against SARS-CoV-2 main protease receptor for the treatment of COVID patients. Similarly, five drug-like candidates were identified using an FBDD technique for treating breast cancer patients by targeting DNA methyltransferase protein [17]. In light of these findings, we propose a hierarchical workflow of computational fragment-based drug designing, the binding free energy calculations, and dynamic simulation approaches for designing molecules against RET proteins. Moreover, no study has been reported to date to identify drug-like molecules using a computational fragment-based drug designing strategy against RET proteins. Therefore, we believe that this fragment-tailoring strategy will provide highly potent drug-like candidates against RET oncogenic drivers for the treatment of NSCLC patients.

## 2. Materials and Methods

### 2.1. Dataset Preparation

A library of 18 compounds with experimental anti-RET activity ranging between 0.4 nM and 200 nM were manually compiled from the literature [18,19]. The list of compounds, class of compounds, and their corresponding inhibitory activity are reported in Supplementary Materials Table S1. The existing 18 RET inhibitors were manually compiled from the literature. The selected molecules cover diverse chemical classes namely benzenoids, diazinanes, indoles, organooxygen, pyrazoles, pyridines, and quinolones. Indeed, the diversity of the selected compounds is likely to be useful in order to generate a broad spectrum of breed molecules [20]. The 3D spatial data file of these compounds was retrieved from the PubChem library [21]. Subsequently, the LigPrep module of Schrödinger was employed for refining the ligand library, such as the inclusion of hydrogen bonds, generation of stereoisomers, and the identification of ionization state. The 3D crystal structure of the RET protein with 2.5Å resolution (PDB ID: 2IVU) were obtained from Protein Data Bank mainly due to the existence of phosphorylated wild type RET kinase domain bound with the existing MKI vandetanib. Note that the A-loop phosphorylated basal state of tyrosine kinase domain stimulates high catalytic activity by regulating cis-autoinhibition. On the contrary, the non-phosphorylated state results in interference of the A-loop conformation with the substrate binding sites. This depicts that a study using phosphorylated RET kinase domain would provide the results with high precision [22]. Thus, we selected the RET protein (PDB Code: 2IVU) for our investigation.

The protein preparation wizard was employed to prepare the RET receptor by eliminating impurities and water molecules, including ionization and hydrogen bonds in the protein structure [23]. Finally, the refined ligand library and the target protein molecule were optimized using the OPLS_2005 force field [24]. The pralsetinib was considered as a control for our analysis as it is an only RET-specific inhibitor that is approved by the Food and Drug Administration (FDA) in the recent time [25,26].

### 2.2. Fragment Tailoring Strategy

This approach works on the principle that merging distinct fragments will result in a new molecule with a binding affinity equal to the sum of each fragment’s specific interactions [16]. A fragment tailoring process was performed with the retrieved known inhibitors of RET using the Schrödinger suite. This process involves the fragmentation of ligand molecules into smaller pieces and the sequential combining of these into novel chemical moieties. The fragmentation of ligand molecules was carried out using fragment.py coding script that was obtained from the Maestro interface. The hybrid molecules were then generated by identifying the overlapping bonds and swapping either side of the fragments. The default parameters such as: (i) the maximum atom-atom distance was 1 Å and (ii) the maximum angle was 15 degrees were considered during breeding process [24]. The overall hybrid molecule or linked fragment (LF) generation process was carried out using the ‘BREED’ facility provided by the Schrödinger suite.

### 2.3. Hybrid Molecule Screening upon RET Target

The refined PDB structure of RET was considered directly for the generation of the receptor grid. In the present study, the scaling factor was fixed as 1.0 and the partial charge cut off was set to 0.25 to soften the non-polar parts of the protein using the Receptor Grid Generation module that is available in the Schrödinger suite. The centroid of the interaction grids was defined by considering the bound ligand molecule. Towards the end, extra precision docking of all the hybrid molecules was performed using the Glide module of the Schrödinger suite. The symmetry-corrected RMSD of the docked posed to its respective input ligand structures were also noted using the “Compute RMSD to input ligand geometries” option that is available during extra precision docking [27].

### 2.4. Rescoring Validation of Hybrid Molecules

Random forest (RF) score is a machine learning-based rescoring strategy that is widely used to validate the ligand and the protein interaction. A folder containing the PDB structure of protein and the ligands serves as input for the rescoring process. This approach employs a random forest algorithm to calculate the RF score based on the atomic distance (less than 12 Å) in the protein-ligand complex. The higher value of the RF score indicates the stronger binding affinity of a ligand towards the protein and vice versa [28]. In the current investigation, the RF score of each protein-ligand complex was generated using RF-Score-VS 1.0 python script that is available at https://github.com/oddt/rfscorevs (accessed on 26 January 2022).

### 2.5. Binding Free Energy Analysis

With the aim of further exploring the interaction of protein-ligand complexes, the binding free energy and the ligand strain energy were calculated using Prime Molecular Mechanics—Generalized Born Model and Solvent Accessibility (MM-GBSA) of Schrödinger. This simulation process employs contributions from rotamer search algorithms, OPLS_2005 force field and VSGB solvent model [29]. The binding free energy was estimated in kcal/mol using the below equation:

ΔG_bind_ = E_complex_ − E_RET_ − E_ligand_ (1)

where ΔG_bind_ is the binding free energy, E_complex_ represents the energy of the complex system, and E_RET_ and E_ligand_ denotes the energy of the protein and unbound ligand, respectively.

### 2.6. HOMO—LUMO Theory

Density functional theory (DFT) analysis was performed to assess the electron transport potential and the electronic properties of the lead molecules. The calculations were performed by using the Jaguar module of Schrödinger. The frontier molecular orbitals including the highest occupied molecular orbital (HOMO) and lowest unoccupied molecular orbital (LUMO) of the ligands were calculated to provide extensive information on electron density clouds. The energy gap was calculated using the estimated HOMO—LUMO values to determine the anti-cancer reactivity of the compounds towards the RET protein. The decreased energy gap of lead compounds demonstrates the better anti-cancer property of the compound and vice-versa [30]. Similarly, the ionization potential (IP) and electron affinity (EA) of the selected compounds were determined during the analysis. The other global descriptors hardness (η), softness (S) and chemical potential (χ, −µ) were calculated using the below equations to determine the reactivity and stability of the compound [31,32]:

(2) $$\mathrm{Hardness}\;\left(\eta \right)=\frac{\mathrm{IP}-\mathrm{EA}}{2}$$

(3) $$\mathrm{Softness}\;\left(S\right)=\frac{1}{\eta }$$

(4) $$\mathrm{Chemical}\;\mathrm{Potential}\;\left(\chi \right)=-\mu =-\frac{\mathrm{IP}+\mathrm{EA}}{2}$$

### 2.7. Drug Likeness and Toxicity Analysis

In the present study, the QikProp module of Schrödinger was implemented to assess the pharmaceutically relevant pharmacokinetic, physiological, and physiochemical properties of the compound including the number of hydrogen bond donors, hydrogen bond acceptors, the predicted octanol or water partition coefficient, the predicted aqueous solubility, the solvent-surface accessibility area, and the blood-brain partition coefficient [33]. In addition, the toxicity profile of the compounds was computationally predicted using ProTox-II software.

### 2.8. Stability and Flexibility Assessment of Hybrid Molecules

Molecular dynamics (MD) simulations of naïve RET proteins and RET-ligand complexes were performed using GROMACS 2018 tool to evaluate the stability and flexibility of complexes in a precise bilayer and hydration environment. We used the GROMOS96 43a1 force field on an NVIDIA DGX workstation for this purpose. A PRODRG server was used to generate the ligand topology files. The prepared protein-ligand complexes were solvated in a dodecahedron box with a volume of 7.66 nm^3^. The box was configured with simple point charge waters and eight chlorine counter ions to neutralize the total system charge. Subsequently, the energy of the complex system was minimized by removing the weak Van der Waal linkages using the Steepest Descent algorithm. Simultaneously, the electrostatic and hydrogen bonds were constrained using the Particle-Mesh Ewald methods and LINCS algorithm. Initially, the position of the complex was restrained using the constant NVT (number of particles, volume, and temperature) phase of equilibration with each step of 2 fs. Later, the system was equilibrated using the NPT phase (constant particle, volume, and temperature) at 300 K with the pressure of 1 bar and lapsing time of 0.1 ps using the Berendsen temperature coupling method. The final simulation step of apo-protein and protein-ligand complex was carried for 200 ns [34,35]. The trajectory of the final step was saved for each 2 fs. The results of the simulation were evaluated using root mean square deviation (RMSD), root mean square fluctuation (RMSF), hydrogen bond linkages, and the free energy landscape using GROMACS utilities.

### 2.9. Compound Reactivity Analysis Using PaccMann and MM-PBSA Analysis

Despite the huge investments that are made for drug development against cancer, it has been reported that 97% of the drug-like molecules have failed in clinical trials due to low target efficacy and off-target toxicities. Hence, a prediction of the compounds activity with high accuracy has become essential. In the current investigation, the sensitivity of the compounds was evaluated using a multimodal neural network-based tool named ‘PaccMann’. This tool utilizes the key pillar information of the compounds such as SMILES sequence, prior information on the intracellular interactions, and gene expression profiles of tumors to predict the sensitivity of compounds against various cancer cell lines with high accuracy [36].

In essence, the empirical binding free energies between the target RET receptor and the hybrid molecules was calculated with the aid of equilibrated trajectory information through molecular mechanics energies that were combined with the Poisson–Boltzmann and surface area continuum solvation (MM-PBSA) strategy [37]. It is important to note that MM-PBSA protocols achieved reasonable correlation with experimental affinities at a much lower computational cost than the other available methods.

### 2.10. Synergism Analysis of Parent Compounds

In order to analyse the synergistic effect of the parent compounds, the simultaneous docking of two compounds against the RET protein was performed using AutoDock 4.2.6. The protein was prepared by adding the hydrogen bonds and Kollman charges. On the other hand, the ligands were prepared by expanding the torsions root and by assigning Gasteiger charges. The prepared molecules were saved in PDBQT format for further analysis. The induced-fit docking of the lead and the reference compounds were performed by setting the grid for the entire macromolecule. Later, the synergistic effect of the compounds against the RET protein was analyzed using a sequential docking procedure with a default centre grid box of x centre = 0.251, y centre = 0.587, and z centre = −1.361, respectively. The binding score of the docked complexes was calculated using the Lamarckian Genetic algorithm [38].

## 3. Results and Discussion

### 3.1. Fragments Tailoring and Docking Analysis

Fragment-based drug designing plays a vital role in the pharmaceutical industry in the identification of novel drug-like molecules with less molecular weight and high chemically diversity in nature [39]. Here, 335 fragments were generated from the 18 known inhibitors of RET for our investigation. These fragments were conjugated using the BREED module of Schrödinger that yielded 115 hybrid molecules. Further, the generated hybrid compounds were subjected to extra precision docking with pralsetinib as the reference molecule. This screening strategy yielded a total of 37 compounds with better binding affinity than pralsetinib (−7.79 kcal/mol). Subsequently, the symmetry-corrected RMSD of the docked poses to its respective input ligand structures was investigated using the Glide module of Schrödinger. It is evident from the literature that the heavy-atom RMSD of the docked poses is considered as a docking success if the pose RMSD is less than 2.0 Å from the input ligand structures [40]. In the present investigation, the RMSD value of the hybrid molecules ranged between 0.218 Å and 10.779 Å, respectively. About 68 out of 115 hybrid molecules were found to have an RMSD value that was less than 2.0 Å. It is to be noted that all the compounds with better binding affinity also showed a satisfactory RMSD value depicting the successful docking pose of the designed ligand against the RET protein.

### 3.2. Rescoring with Machine Learning Algorithm

The RF score is a novel machine learning-based strategy that is widely used to re-score the binding affinity between the protein and the ligand molecule [34]. Although the RF scoring method is less precise on physiochemical features, it transcended the existing conventional scoring system to determine the binding affinity [41]. Thus, in the present study, the RF-scoring system was employed to rescore all the hybrid compounds that were obtained during the breeding process. The results indicate that 82 out of 115 compounds have a higher score than pralsetinib (RF score = 5.962). The docking and ML results are integrated to identify highly effective compounds and eliminate the false positive designs. Overall, 23 compounds possess both better binding energy and RF score than pralsetinib. The results are presented in Table 1.

### 3.3. Binding Free Energy Calculations

The relative binding affinity of the ligands towards the RET receptor was calculated using the Prime module of Schrödinger. The overall binding free energy of ligands varied between −78.895 kcal/mol and −30.081 kcal/mol (Table 2). Eventually, the only five compounds showed a significant ΔG_bind_ value that was greater than pralsetinib (−63.348 kcal/mol). Among the contribution of different energy terms, the Van der Waals force of all the compounds was found to have a major contribution and facilitated the strong binding towards the RET protein. The electrostatic potential exhibited the second highest contribution for binding affinity; however, high solvation energy significantly nullified the effect of the electrostatic potential to ΔG_bind_. The lower value of covalent interaction in all five compounds denotes the high thermostability characteristics of the molecules. Particularly, it indicates the stabilized association with the RET protein to a larger extent. In addition, the ligand strain energy of the compounds was evaluated to assess the deformation of the ligands during interaction [42]. It is evident from Table 2 that the top five compounds undergo less deformation than the reference molecule during the interaction with the RET receptor. Although LF3 showed the highest docking score, the decrease in binding free energy during MM-GBSA analysis is due to increased ligand strain energy at the time of binding. Thus, the five hybrid molecules namely LF1, LF2, LF21, LF27, and LF88 showed satisfactory energy contributions and less ligand strain during the interaction with the RET protein, and so were screened for further analysis.

### 3.4. Frontier Molecular Orbital Analysis

**Figure 1:**
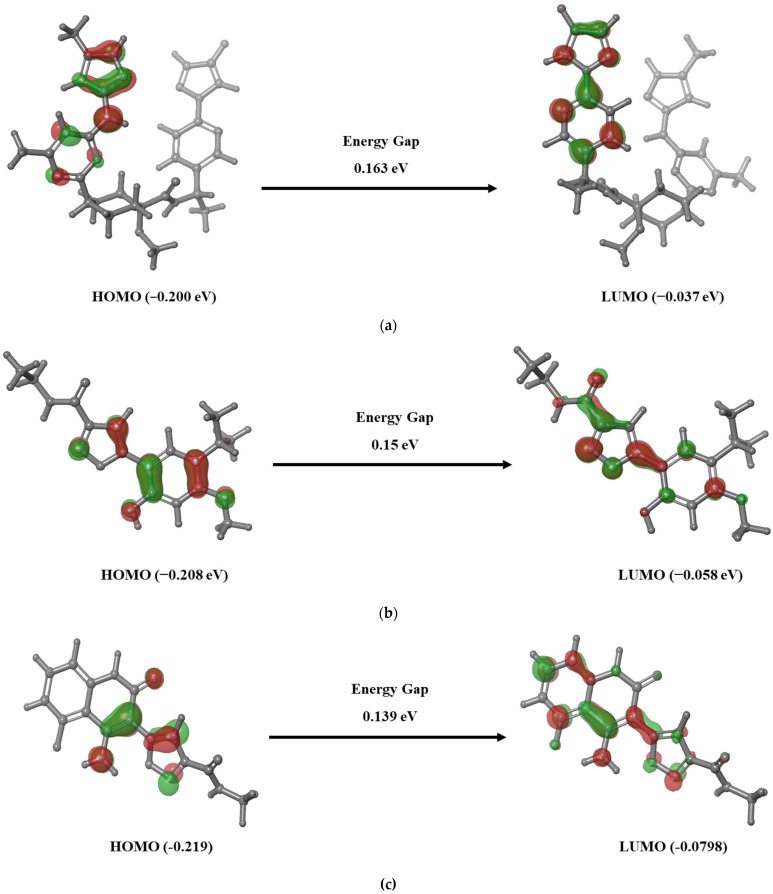

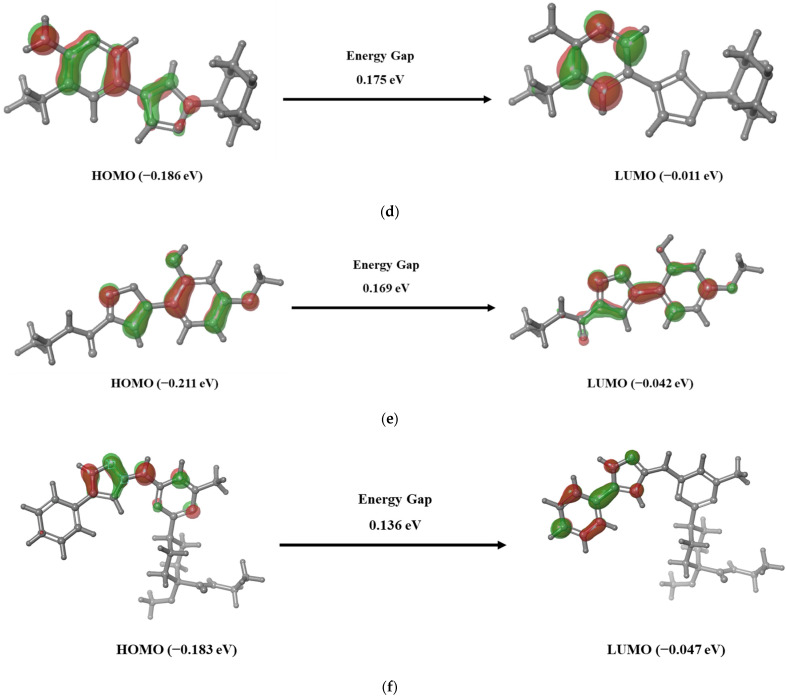
Chemical reactivity analysis of the linked fragments using density functional theory analysis: (**a**) Pralsetinib, (**b**) LF1, (**c**) LF2, (**d**) LF21, (**e**) LF27, and (**f**) LF88.

Estimating the energy gap between the frontier molecule orbitals i.e., HOMO and LUMO plays an important role in analyzing the chemical reactivity of the compounds. An E_HOMO_ represents the ionization potential as the molecule easily loses its electron at its energized state, whereas E_LUMO_ represents the electron affinity as the molecule accepts the electron. The E_HOMO_ and E_LUMO_ of the reference and hybrid molecules LF1, LF2, LF21, LF27, and LF88 were calculated and are presented in Figure 1. The positive and negative electron density in the HOMO-LUMO plot is represented in red and green, respectively. It is observed that LF1, LF2, and LF88 have less energy gap than LF21 and LF27, depicting the highly favorable potential reactions against RET. These findings imply that the hybrid molecules LF1, LF2, and LF88 will exhibit higher chemical reactivity. The literature shows that the higher HOMO energy than LUMO of a compound demonstrates the ability to donate electrons to the partner receptor binding site. Additionally, it depicts the favorability of forming a hydrogen bond between the ligand and the protein [43]. Interestingly, the results suggests that the HOMO of all selected ligands was higher than the LUMO, depicting the ability of the ligands to donate electrons and also that they had favorable hydrogen bond formation with the RET binding site residues.

Consequently, the ionization potential, electron affinity, hardness, softness, and electronegativity of the reference and the selected molecules were calculated to determine the chemical reactivity and stability of the compounds. It is evident from our results (Table S2) that the ionization potential of all the compounds is higher than the electron affinity, indicating the better electron donating capability of the hybrid molecules. It is evident from the results that the developed hybrid molecules except for LF21 and LF27 were highly reactive with a high hardness (~4.0) and low softness (~2.0). The lower chemical potential of LF1 (3.97), LF2 (4.88), and LF88 (−4.41) demonstrates the unwillingness for dissociating into its elements and its stability. On the other hand, a higher negative chemical potential of LF21 (2.95) and LF27 (3.437) shows the lower stability than the other compounds. The preceding results depict that the investigated molecules LF1, LF2, and LF88 are chemically hard molecules with high electron donating capability and kinetic stability.

### 3.5. Interaction and ADMET Analysis

**Figure 2:**
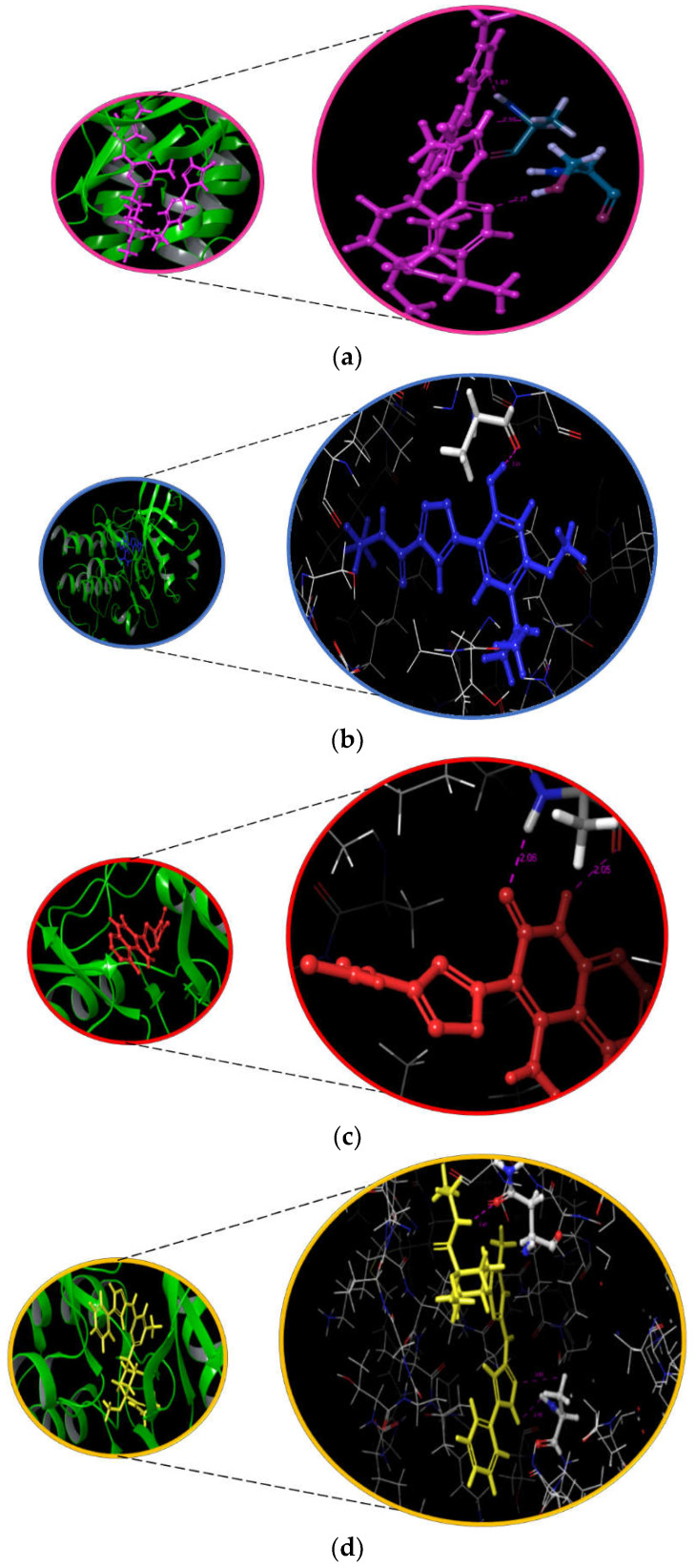
The interaction pattern of the reference molecule (**a**) Pralsetinib and hybrid molecules (**b**) LF1, (**c**) LF2, and (**d**) LF88 with the RET protein.

The interaction pattern of the hybrid molecules to the binding pocket of RET is represented in Figure 2 and Table S3. Additionally, the 3D image of the docked RET-ligand complexes is represented in Figure S1. The ligand interaction diagram of pralsetinib clearly shows the formation of two hydrogen bonds between the ALA807 residue of the RET and the carboxamide group. In addition, one more hydrogen bond was found between the pyridine ring of pralsetinib and SER811 of RET. The literature evidence also highlights the contribution of residues in the binding pocket of RET [44]. On analyzing the binding pattern of LF1, one hydrogen bond was found between the hydroxy group of LF1 and ALA807 residue of the protein. In the case of LF2, a two hydrogen bond formation was observed between ALA807 and NH/O of the azaarene group. Likewise, a two hydrogen bond formation was observed between N/NH of the azaarene functional group of LF88 and ALA807. In addition, a one hydrogen bond formation was found between the NH group of LF88 and the key hydrophobic residue ASN879 of RET. These results are evident that the designed fragments able to replicate the binding pattern to that of pralsetinib. In addition, the carboxamide and azaarene functional groups that were identified in the hit molecules were reported recently to have anticancer activity [45,46].

The ADMET analysis of the compounds was performed using the QikProp module of Schrödinger and ProTox-II software to prevent the elimination of molecules during clinical trials. The results in Table 3 indicate the satisfactory pharmacodynamic, pharmacokinetic, and toxicity values for all the three hit hybrid compounds. Specifically, the important central nervous system response that was stimulated by the hit compounds was almost similar to pralsetinib. Undeniably, the human oral adsorption of LF1 and LF2 was higher than pralsetinib, indicating the higher efficacy of the molecules that can be easily attained via oral administration. Moreover, all the lead compounds belonging to class 5 toxicity with LD50 ranged from 2000 to 5000. For instance, the LD50 of LF1, LF2, and LF88 was found to be 4000 mg/kg, 2500 mg/kg, and 3500 mg/kg, respectively, which is less toxic than pralsetinib as it showed an LD50 value of 800 mg/kg. The higher the value of LD50, the less harmful the compounds will be in humans [47]. Thus, all the designed compounds were less harmful to humans than pralsetinib.

### 3.6. Molecular Dynamics Simulation

#### 3.6.1. Stability Analysis of Complex System

**Figure 3:**
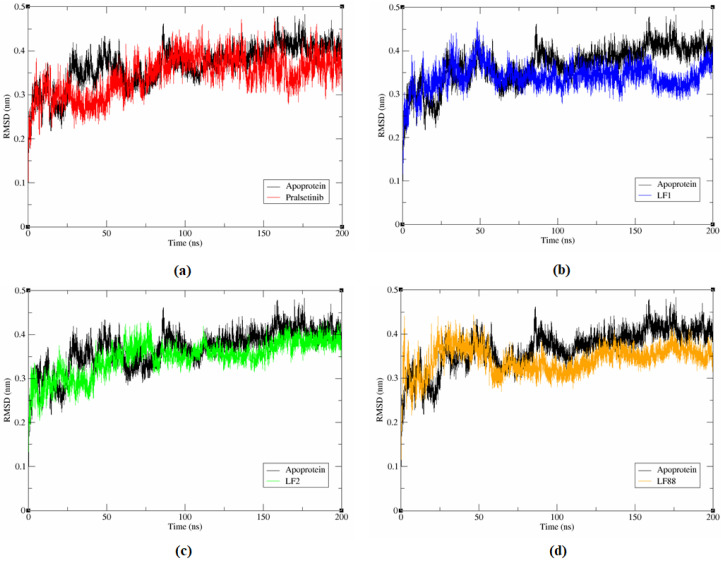
Time dependent MD simulation of apoprotein and protein-hybrid molecule complexes: (**a**) Pralsetinib, (**b**) L1, (**c**) LF2, and (**d**) LF88.

The molecular dynamic simulations for the RET apoprotein and the protein-ligand complex was analysed for 200 ns using the GROMACS 2018 tool to understand the stability, structural details, and conformational behaviour of the protein-ligand complexes. RMSD plots are used to analysis the extent of deviation of atoms in this study. It is evident from Figure 3 that an increased deviation was observed until 50 ns on the binding of compounds, including pralsetinib with the RET protein. Consequently, all the hybrid molecules maintained equilibrium between 50 ns and 150 ns except for pralsetinib, which exhibited an increased deviation until 100 ns. Towards the end of 200 ns of simulation, a minimal RMSD value of 0.370 nm, 0.361 nm, and 0.331 nm was observed among the RET-LF1, RET-LF2, and RET-LF88 complexes, respectively, smaller than apoprotein (0.414 nm) and the RET-pralsetinib complex (0.385 nm). The results are well correlated with our initial analysis of docking and binding free energy.

The lower RMSD value of the designed compounds than the apoprotein and RET-pralsetinib complex suggests the stable binding of ligands (LF1, LF2, and LF88) than pralsetinib in the binding pocket of the RET receptor. The literature evidence highlights that a RMSD value of less than 0.4 nm is certainly needed for the ligand to stay within the binding pocket of the protein [48]. It is worth mentioning that the RMSD values of the designed compounds ranged from 0.3 nm to 0.4 nm. Thus, we hypothesize that the developed three hybrid molecules could serve as an active inhibitor against RET.

#### 3.6.2. Flexibility Analysis of Complex System

**Figure 4:**
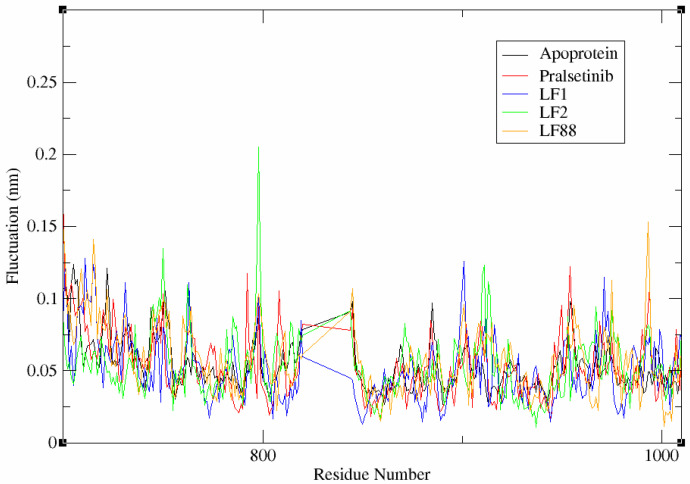
Fluctuation analysis of the apoprotein and complex systems during simulation process.

The RMSF plots analyse the mobility and fluctuation of residues within the RET-ligand complexes. The trajectories are shown in Figure 4. It is evident from the figure that a similar pattern of fluctuation was observed among all the RET-ligand complex systems. A minimal deviation of less than 0.05 nm was observed for the crucial residues, such as ARG770 and GLY810 of the RET protein during the binding of hybrid molecules. It is to be noted that the stability of the RET-ligand complex is due to the formation of hydrogen bonds between these residues and the ligands. Prominently, the important residue ALA807, that is also found within the conserved interaction region, showed minimal fluctuation of ~0.02 nm. On the other hand, the residues PRO992–LYS994 exhibited high fluctuation of 0.1 nm in all the protein-ligand complex systems. In the case of LF2, a high fluctuation of ~0.2 nm was observed, even at GLY798 residue during RMSF analysis. This evidence highlights the identified residues PRO992–LYS994 and GLY798 as unfavorable regions for the interaction of ligands with RET. Overall the RMSF value of the aMD simulation differentiates the terminal of the complexes as an organized region or a loosely structured region. A lower RMSF value indicates an organized end terminal, whereas a higher RMSF value denotes a loosely structured terminal end of the complex system [49]. On analyzing the fluctuation data of the apoprotein and the complex systems RET-pralsetinib, RET-LF1, LF2, and LF88, values of 0.0696 nm, 0.0690 nm, 0.0799 nm, 0.1103 nm, and 0.0539 nm, respectively, were observed. The results significantly shows that all the designed ligands contain organized terminal ends. This facilitates the convenient positioning of the ligands within the binding pocket of RET during the interaction than pralsetinib.

#### 3.6.3. Hydrogen Bond Interaction Analysis

**Figure 5:**
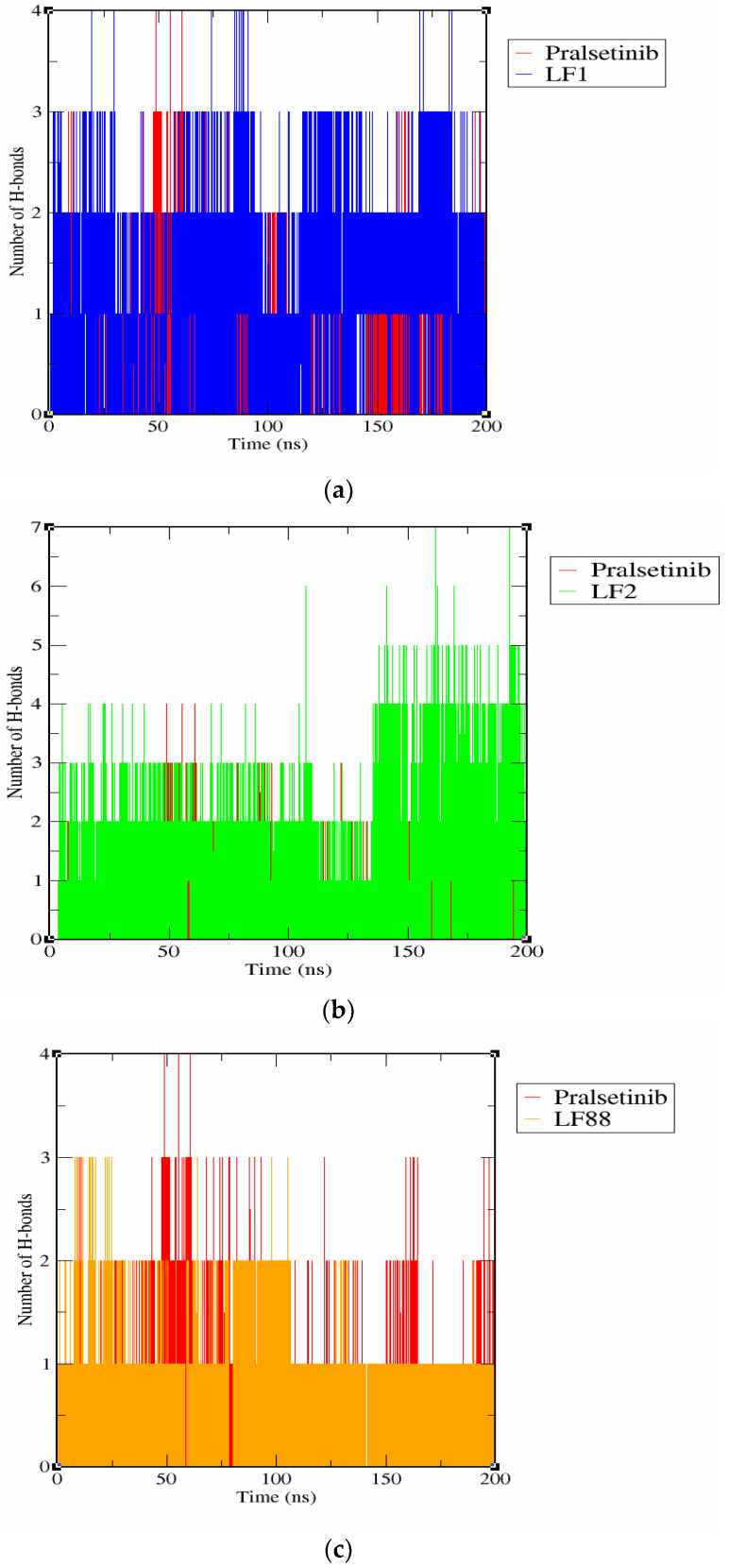
Comparative analysis of the hydrogen bonds within 0.35 nm of the protein-ligand system: (**a**) Pralsetinib and LF1, (**b**) Pralsetinib and LF2, and (**c**) Pralsetinib and LF88.

In general, the stability of the complex system depends on the type of transient interactions including Van der Waals force, hydrogen bond interaction, electrostatic interaction, and many other forces. Among them, the formation of a hydrogen bond between the protein and the ligand is considered as the most important transient force that is responsible for the stability of the protein-ligand complex system [28]. Thus, the number of hydrogen bonds in each protein-ligand interaction was estimated using the trajectory files of the MD simulation. We can observe from Figure 5 that RET-pralsetinib, RET-LF1, RET-LF2, and RET-LF88 complexes were found to exhibit 0–4, 0–4, 0–7, and 0–3 hydrogen bond interactions, respectively, during the simulation. These results highlight the stable binding of the hybrid molecules to RET than pralsetinib.

#### 3.6.4. Free Energy Landscape

**Figure 6:**
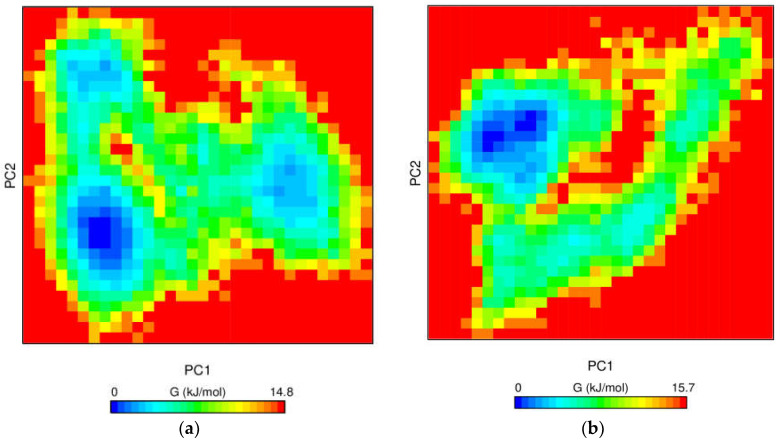

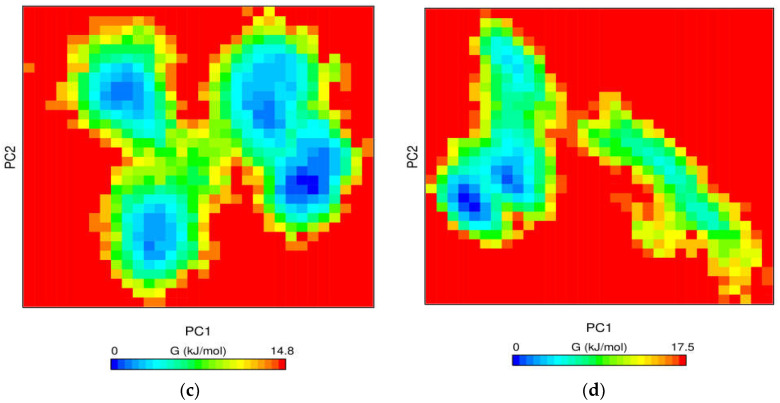
Contour plot of the free energy landscape analysis: (**a**) RET-Reference complex, (**b**) RET-LF1 complex, (**c**) RET-LF2 complex, and (**d**) RET–LF88 complex.

The conformational changes of the protein-ligand complex were further evaluated using the GROAMCS tool gmx_sham. Gibbs free energy measures the exchange of heat in a closed protein-ligand system [50]. During this process, the molecular fluctuation and the energy minima conformation of the protein-ligand complex was estimated. Initially, the gmx_covar tool was implemented to calculate the covariance matrix containing the eigenvalues. Later the matrix was diagonalized to produce eigenvectors. At the end, the two principal components, PC1 and PC2, of the ligands, as represented in Figure 6, were obtained using the gmx_anaeig tool [51]. The color gradient from blue to yellow represents the energy minima favoured to the unfavored conformation of the complex. The hybrid compounds LF2 and LF88 exhibited one deep well energy basin that was similar to pralsetinib. On the other hand, two deep energy basins were observed in the case of LF1. The Gibbs free energy of LF1 and LF2 was found to be 15.7 kJ/mol and 14.8 kJ/mol, respectively, which were almost equivalent to the Gibbs free energy of pralsetinib. Nevertheless, LF88 exhibited a slightly higher Gibbs free energy of 17.5 kJ/mol during the interaction. From Figure 6, it is apparent that the energy basins were distinct, clear, and broad in all three protein-hybrid complexes. Importantly, the results are similar to the Gibbs free energy of pralsetinib, illustrating the stable conformation of the complexes.

### 3.7. In Silico Compound Activity Analysis

The literature evidence highlights that the MM-PBSA calculation and deep learning-based prediction systems have dramatically improved the ability to simulate complex processes computationally. The MM-PBSA protocols have achieved reasonable correlation with experimental affinities at a much lower computational cost than the other methods [52]. Slynko et al. observed a correlation coefficient of 0.78 between the experimental pIC50 and the computational MM-PBSA binding affinities using a PRK1 inhibitors data set [53]. On the other hand, the deep learning model, PaccMann algorithm, is an effective validation toolbox in the drug-repurposing approach which displayed the R2 value of 0.86 and RMSE value of 0.89. These coefficients highlight the strong correlations between the resultant data and experimentally-determined results [54].

In the current investigation, the compounds activity was examined against the LC-2/ad cell line using the PaccMann algorithm. The results are reported in Table S4. It is evident from the table that hit compound LF2 and LF88 exhibited better inhibitory activity to that of the reference molecule, pralsetinib, while LF1 showed comparatively less activity in our analysis. Altogether, the compounds activity was further validated using MM-PBSA analysis and the results are shown in Table 4. All the three hybrid molecules exhibited less binding energy with more stable conformation than pralsetinib. The overall binding free energy of RET-pralsetinib, RET-LF1, RET-LF2, and RET-LF88 were found to be −9.445 ± 65.091 kJ/mol, −15 ± 22.651 kJ/mol, −13.158 ± 16.317 kJ/mol, and −29.627 ± 27.501 kJ/mol, respectively. The overall binding energy included contributions of electrostatic energy, Van der Waals energy, SASA energy, and polar solvation energy. Van der Waals energy was found to be the major contributor of total binding energy among all the RET-ligand complexes. This observation correlates well with our MM-GBSA analysis. From the view of this evidence, we anticipate that this work will provide a meaningful perspective for experimental biologists and support more progress in this cancer therapeutics field.

### 3.8. Synergistic Effect Analysis

The parental compounds of all the three hybrid molecules were identified to evaluate the synergistic effect against the RET receptor. Sequential docking was performed for the parental compounds of all the hybrid molecules using AutoDock 2.5.6. The binding affinity was calculated for the parental drugs: (i) LOXO-292 and Luminespib, (ii) Dovitinib and Luminespib, and (iii) Pralsetinib and Luminespib. It is worth noting that the fragment of luminespib drug is present in all the three-hybrid molecules. Moreover, luminespib is an FDA-approved heat shock protein-90 inhibitor with antineoplastic activity [55]. Hence, the synergistic effect analysis of these parental compounds will provide valuable insight in developing a novel RET inhibitor. Molecular docking of pralsetinib individually showed a binding energy of −3.39 kcal/mol. However, a substantial improvement of the binding energy of −5.38 kcal/mol, −4.06 kcal/mol, and −3.84 kcal/mol, respectively, were observed on the binding of two drugs simultaneously with RET (Table 5). The binding mode of the docked ligand molecules with RET are shown in Figure S2. It is evident from our results that the reactivity of combining the parental drugs is significantly greater than pralsetinib. Hence, we are certain that the fragments that were generated using these parental compounds might exhibit better efficacy in experimental analysis.

### 3.9. Evaluation of Hybrid Compounds against Mutant RET

Drug resistance that is due to point mutations is the major hindrance to developing novel compounds against RET in the drug discovery process. Specifically, the emergence of solvent front mutations and gatekeeper mutations including at the position G810, M918, V804, and Y806 prevents the drug from accessing the binding pocket of RET [56,57]. Here, we performed docking studies against all the mutant RET structures to explore the hybrid molecule activity. The binding affinity of compounds against the mutant RET is represented in Table 6. About 11 distinct RET mutant structures consisting of four solvent front mutation, four gatekeeper mutations, and three other mutations were generated using a homology modelling process. Among the developed hybrid molecules, LF2 and LF88 had overcome seven-point mutations including two solvent front mutations (G810C and G810R); three gatekeeper mutations (V804L, V804M, and Y806C/N); and two other mutations (M918T and V738A) with higher binding affinity. In the case of LF1 and pralsetinib, LF1 had overcome six-point mutations against RET. On the contrary, pralsetinib showed inhibitory activity against three of the investigated mutants of the RET protein. These data depict the potency of hybrid molecules to overcome drug resistance in RET-positive NSCLC patients.

## 4. Limitations and Future Prospective

The bottleneck among RET inhibitors is acquired drug resistance, which results in decreased therapeutic effectiveness in NSCLC patients. Hence, we tested the efficacy of hybrid compounds against 11 distinct RET mutations. Despite the fact that the developed linked fragments have a substantial effect against the solvent front and gatekeeper mutations, experimental confirmation of the hybrid molecule using mutant cell lines is required to verify these findings. Moreover, the toxicity investigations of these molecules, either using an in vivo micronucleus assay or an in vitro genotoxicity assay, are also promising in future directions.

## 5. Conclusions

A fragment-based drug-designing strategy was implemented in the present study to develop the novel inhibitory compounds against the RET protein. The hybrid molecules were probed for their binding affinity and drug-likeliness property using molecular docking with native and mutant RET kinase domain. About 33 hybrid compounds exhibited a better binding affinity than pralsetinib. Further, the docking process was validated using two different methods such as RF-Score and Prime MM-GBSA, resulting in five novel hybrid compounds with a better binding free energy than the reference molecule. DFT calculations yielded three linked fragments LF1, LF2, and LF88 with better chemical reactivity with RET. The study also highlights the chemical reactivity, electron donating capability, and the stability of the hybrid molecules. The molecular dynamic simulation of these lead molecules portrays the existence of a more stable conformation in the binding pocket of RET protein than pralsetinib. The resultant compounds also exhibited a satisfactory binding energy profile against multiple RET variants. Finally, the synergistic effect of the parent compounds together with MMPBSA and PaccMann analysis provides better clues of the linked fragments activity against the RET protein. Based on these findings, we hypothesize that the administration of luminespib either with LOXO-292 or with pralsetinib or dovitinib may provide a better cure against the different RET variants. Indeed, our research findings will aid in the development of new molecules with commercial value in the near future.

## Figures and Tables

**Table 1:** The drug-likeliness and binding affinity analysis of the RET-linked fragment complexes using docking and machine learning strategies.

S. No.	Control and Linked Fragments	Stars	XP gScore (kcal/mol)	RMSD Å	RF-Score
1	Pralsetinib	0	−7.79	6.440	5.962
2	LF1	0	−8.2	0.257	6.334
3	LF2	0	−8.885	0.558	6.108
4	LF3	0	−11.039	0.535	6.397
5	LF4	0	−9.505	1.437	5.967
6	LF5	0	−7.817	0.717	5.968
7	LF6	0	−7.947	8.341	5.967
8	LF7	0	−8.134	0.304	6.135
9	LF8	0	−9.198	6.178	6.071
10	LF9	0	−9.19	1.144	5.973
11	LF10	0	−8.638	0.423	6.09
12	LF11	0	−8.25	0.798	5.976
13	LF12	0	−8.375	0.528	6.044
14	LF13	0	−8.257	0.733	5.967
15	LF14	0	−8.638	1.167	5.983
16	LF15	0	−9.439	7.719	5.976
17	LF16	0	−7.972	8.395	6.15
18	LF17	0	−9.577	1.175	5.98
19	LF18	0	−7.797	9.557	6.269
20	LF19	0	−7.985	7.017	6.154
21	LF20	0	−8.621	2.099	5.963
22	LF21	0	−8.828	0.597	5.961
23	LF22	0	−8.3	0.656	5.961
24	LF23	0	−8.17	0.228	5.958
25	LF24	0	−8.695	0.588	5.956
26	LF25	0	−9.611	1.041	5.955
27	LF26	0	−7.852	1.439	5.953
28	LF27	0	−7.913	0.224	5.951
29	LF28	0	−9.133	0.425	5.95
30	LF29	0	−9.835	4.270	5.948
31	LF30	0	−7.777	5.224	6
32	LF31	0	−7.746	0.218	6.2
33	LF32	0	−7.739	2.131	6.141
34	LF33	0	−7.696	5.693	6.116
35	LF34	0	−7.668	1.040	6.045
36	LF35	0	−7.619	0.605	5.983
37	LF36	0	−7.535	1.023	5.978
38	LF37	0	−7.526	0.564	5.983
39	LF38	0	−7.491	4.930	6.139
40	LF39	0	−7.49	0.329	6.085
41	LF40	0	−7.48	8.155	5.952
42	LF41	0	−7.468	1.141	5.973
43	LF42	0	−7.467	0.801	6.284
44	LF43	0	−7.458	0.789	5.965
45	LF44	0	−7.437	1.432	5.971
46	LF45	0	−7.421	3.320	5.955
47	LF46	0	−7.41	0.814	5.997
48	LF47	0	−7.389	1.163	6.102
49	LF48	0	−7.355	0.326	5.955
50	LF49	0	−7.339	2.510	5.976
51	LF50	0	−7.322	0.431	5.974
52	LF51	0	−7.289	6.173	5.97
53	LF52	0	−7.283	0.695	5.966
54	LF53	0	−7.207	3.886	6.215
55	LF54	0	−7.201	1.094	5.962
56	LF55	0	−7.199	1.717	6.123
57	LF56	0	−7.186	4.928	5.971
58	LF57	0	−7.139	1.687	6.114
59	LF58	0	−7.137	0.482	5.963
60	LF59	0	−7.087	0.569	6.087
61	LF60	0	−7.064	5.467	5.963
62	LF61	0	−7.014	0.255	5.963
63	LF62	0	−7.01	5.968	6.086
64	LF63	0	−6.992	0.257	5.957
65	LF64	0	−6.86	4.837	6.093
66	LF65	0	−6.835	4.914	5.974
67	LF66	0	−6.744	0.960	5.958
68	LF67	0	−6.72	0.984	5.97
69	LF68	0	−6.633	5.297	5.952
70	LF69	0	−6.603	0.990	5.985
71	LF70	0	−6.598	1.021	5.971
72	LF71	0	−6.535	1.020	5.99
73	LF72	0	−6.434	6.628	5.959
74	LF73	0	−6.424	6.309	6.117
75	LF74	0	−6.401	3.922	5.967
76	LF75	0	−6.375	0.630	6.011
77	LF76	0	−6.188	1.425	5.962
78	LF77	0	−6.094	0.317	6.184
79	LF78	0	−6.052	2.328	5.982
80	LF79	0	−5.865	3.356	6.172
81	LF80	0	−5.69	4.500	5.971
82	LF81	0	−5.583	3.759	6.036
83	LF82	0	−5.511	6.635	6.133
84	LF83	0	−5.344	2.498	5.95
85	LF84	0	−4.901	4.213	5.957
86	LF85	0	−4.717	2.020	6.083
87	LF86	0	−4.199	2.131	6.099
88	LF87	0	−3.846	0.665	5.964
89	LF88	1	−8.421	0.912	6.193
90	LF89	1	−8.639	0.666	6.01
91	LF90	1	−7.861	1.884	6.283
92	LF91	1	−9.161	9.304	5.957
93	LF92	1	−7.602	0.326	6.026
94	LF93	1	−6.7	7.458	6.044
95	LF94	1	−6.28	0.958	5.961
96	LF95	1	−6.021	3.630	7.361
97	LF96	1	−5.993	0.593	6.992
98	LF97	1	−5.952	10.779	5.962
99	LF98	1	−5.479	6.19	5.978
100	LF99	2	−7.692	6.267	5.984
101	LF100	2	−7.699	6.585	6.157
102	LF101	2	−8.121	0.669	6.045
103	LF102	2	−9.068	1.397	6.027
104	LF103	2	−8.167	0.789	5.952
105	LF104	2	−5.543	4.837	5.964
106	LF105	4	−6.998	3.025	6.035
107	LF106	5	−6.941	2.617	6.078
108	LF107	5	−3.98	2.962	5.957
109	LF108	5	−6.588	5.214	5.977
110	LF109	6	−4.871	1.902	5.971
111	LF110	6	−5.554	7.447	5.957
112	LF111	7	−6.089	0.562	5.96
113	LF112	8	−6.029	0.938	5.962
114	LF113	8	−5.723	0.491	6.178
115	LF114	8	−8.13	1.367	5.959
116	LF115	8	−6.545	1.339	5.96

**Table 2:** Binding free energy analysis of linked fragments using Prime MM-GBSA approach.

Control and Linked Fragments	dG Bind (kcal/mol)	Van der Waal’s Energy (kcal/mol)	Ligand Strain Energy (kcal/mol)	Electrostatic Potential (kcal/mol)	Covalent Interaction (kcal/mol)	Lipophilicity (kcal/mol)	Solvation Energy (kcal/mol)
Pralsetinib	−63.348	−58.387	6.20432	−12.472	−0.4283	−19.969	37.3355
LF1	−78.875	−55.285	5.209	−33.627	1.512	−21.143	32.186
LF27	−73.11	−60.237	2.678	−21.049	2.615	−25.583	32.147
LF2	−70.752	−47.964	3.018	−24.213	1.891	−18.239	20.196
LF21	−63.654	−54.224	2.992	−19.372	1.127	−24.461	34.469
LF88	−63.521	−46.248	5.237	−20.513	3.478	−24.643	26.701
LF29	−62.965	−45.4	2.562	−25.554	2.113	−22.992	30.221
LF3	−62.895	−52.664	6.212	−16.773	−0.312	−19.834	27.54
LF4	−60.017	−39.524	2.804	−21.897	−0.561	−21.251	25.189
LF5	−57.679	−49.011	5.349	−35.466	2.096	−19.2	45.57
LF89	−55.77	−44.968	6.72	−19.658	5.17	−18.602	23.929
LF6	−55.462	−49.877	8.647	−29.366	4.238	−16.002	38.428
LF25	−55.094	−50.213	8.401	−10.118	2.115	−19.658	24.019
LF91	−54.683	−42.063	2.842	−14.307	1.961	−17.836	19.09
LF23	−53.86	−44.928	4.357	−13.386	1.727	−20.908	25.182
LF24	−52.802	−31.177	3.614	−23.708	0.529	−16.921	20.646
LF7	−52.465	−51.814	4.835	−35.392	3.19	−15.905	49.912
LF22	−51.478	−43.117	3.707	−16.363	1.272	−17.922	25.454
LF8	−51.337	−44.893	5.922	−14.472	5.267	−15.919	20.795
LF9	−50.681	−37.001	8.023	−22.027	6.327	−16.065	20.883
LF10	−50.255	−43.526	4.463	−13.965	4.363	−14.628	19.622
LF101	−49.18	−41.558	4.021	−13.997	2.135	−14.383	19.527
LF102	−48.459	−33.805	4.821	−18.293	3.508	−16.351	18.643
LF11	−47.678	−43.294	4.058	−10.555	2.239	−17.1	22.252
LF103	−47.375	−50.103	8.598	−28.738	3.098	−17.355	48.194
LF12	−47.014	−29.869	1.574	−15.849	1.325	−18.546	17.847
LF20	−46.6	−32.975	1.8	−12.629	1.051	−20.039	18.639
LF28	−46.45	−36.202	2.732	−16.141	2.933	−16.087	20.6
LF114	−46.195	−42.487	7.755	−12.376	4.829	−20.4	25.46
LF90	−44.869	−37.752	2.023	−11.832	1.761	−13.851	18.063
LF13	−44.188	−34.743	1.058	−10.255	0.955	−15.561	16.05
LF14	−43.03	−27.483	1.191	−16.22	0.989	−17.025	18.572
LF15	−41.74	−42.888	15.734	−25.959	9.447	−18.956	38.089
LF16	−40.204	−40.209	6.393	−27.114	4.77	−9.159	33.502
LF17	−39.071	−42.268	22.468	−27.647	12.815	−25.637	45.318
LF26	−38.404	−41.666	14.545	−27.751	8.215	−17.893	43.315
LF18	−31.506	−35.323	16.143	−0.157	0.576	−16.869	20.436
LF19	−30.081	−27.423	4.861	−21.575	5.222	−9.01	24.761

**Table 3:** The ADME and toxicity analysis of the lead linked fragments using the QikProp module and ProTox-II server.

Control and Linked Fragments	CNS	HOA	Hepatotoxicity	Carcinogenicity	Immunotoxicity	Mutagenicity	Cytotoxicity	Toxicity Class
Pralsetinib	−2	2	Active	Inactive	Active	Inactive	Inactive	Class 4
LF1	−2	3	Active	Inactive	Inactive	Inactive	Inactive	Class 5
LF2	−2	3	Active	Active	Inactive	Active	Inactive	Class 5
LF88	−1	1	Inactive	Active	Inactive	Inactive	Inactive	Class 5

**Table 4:** MM-PBSA binding free energy calculations of RET-pralsetinib and RET-hybrid molecules complexes.

S. No.	Energy Terms (kJ/mol)	RET-Pralsetinib	RET-LF1	RET-LF2	RET-LF88
1	Binding Energy	−9.445 ± 65.091	−15 ± 22.651	−13.158 ± 16.317	−29.627 ± 27.501
2	Van der Waal’s Energy	−23.022 ± 53.334	−10.678 ± 11.115	−8.669 ± 0.329	−24.053 ± 31.102
3	Electrostatic Energy	−0.074 ± 3.936	−4.928 ± 25.728	−7.359 ± 2.903	−5.523 ± 13.183
4	Polar Solvation Energy	15.905 ± 55.514	3.008 ± 0.268	6.024 ± 1.240	4.021 ± 11.638
5	SASA Energy	−2.254 ± 6.035	−2.402 ± 0.107	−3.154 ± 0.124	−4.072 ± 8.184

**Table 5:** Synergistic analysis of lead compounds against RET protein using AutoDock.

S. No.	Compound 1	Compound 2	Docking Score (kcal/mol)
1	Pralsetinib	Not applicable	−3.39
2	Loxo-292	NVP-AUY922	−5.38
3	Dovitinib	NVP-AUY922	−4.06
4	Pralsetinib	NVP-AUY922	−3.84

**Table 6:** Mutational analysis of the linked fragments using molecular docking analysis.

S. No	Native and Mutant RET Proteins	Glide XP Gscore (kcal/mol)			
		Pralsetinib	LF1	LF2	LF88
1	Native	−7.79	−8.2	−8.885	−8.421
2	G810C	−3.69	−4.035	−4.404	−3.655
3	G810R	−3.356	−5.741	−3.845	−4.842
4	G810S	−6.69	−4.403	−4.404	−3.655
5	G810V	−5.684	−3.97	−4.547	−4.86
6	M918T	−4.85	−6.736	−8.235	−7.647
7	V738A	−4.555	−5.715	−6.457	−5.282
8	V804E	−6.682	−4.114	−4.533	−5.61
9	V804L	−8.067	−5.186	−8.445	−8.412
10	V804M	−7.625	−5.46	−8.337	−7.754
11	Y806C	−5.764	−6.121	−5.572	−6.02
12	Y806N	−4.810	−6.590	−5.685	−6.297

## Supplementary Materials

The following are available online, Table S1: List of the known RET inhibitors with its inhibitory activity. Table S2: Calculated global descriptors of hybrid molecules using LACV3P++** energy level. Table S3: Hydrogen bond interaction involved between the ligand molecules and the receptor. Table S4: In silico sensitivity analysis of control and hybrid molecules against LC-2/ad cell line. Figure S1: 3D visualization of bound protein ligand complexes: (a) RET-pralsetinib, (b) RET-LF1, (c) RET-LF2 and (d) RET-LF88 complex. Figure S2: The synergistic docking of parental compounds where C1, C2, C3, and C4 denotes Luminespib, Pralsetinib, Dovitinib, and LOXO-292, respectively. The white circle indicates the binding position of compound 1 and the blue circle represents the respective binding mode of the 2nd compound.

## Sample Availability

Samples of the compounds are not available from the authors.
