# Supplementary material for: Designing Novel Compounds for the Treatment and Management of RET-Positive Non-Small Cell Lung Cancer—Fragment Based Drug Design Strategy
Source: Molecules. 2022 Feb 28;27(5):1590. doi: 10.3390/molecules27051590 (PMC8911629; doi:10.3390/molecules27051590)
Supplement: Supplementary file 1 [file molecules-27-01590-s001.zip › molecules-1596419-supplementary.pdf]

List of Tables

Table S1: List of known RET inhibitors with its inhibitory activity.

Table S2: Calculated global descriptors of hybrid molecules using LACV3P++** energy level.

Table S3: Hydrogen bond interaction involved between the ligand molecules and the receptor.

Table S4: In silico sensitivity analysis of control and hybrid molecules against LC-2/ad cell line.

List of Figures

Figure S1: 3D visualization of bound protein ligand complexes: (a) RET-pralsetinib, (b) RET-LF1, (c) RET-LF2 and (d) RET-LF88 complex.

Figure S2: The synergistic docking of parental compounds where C1, C2, C3 and C4 denotes Luminespib, Pralsetinib, Dovitinib and LOXO-292 respectively. White circle indicates the binding position of compound 1 and the blue circle represents the respective binding mode of the 2nd compound.

Table S1: List of known RET inhibitors with its inhibitory activity.

S. No	Compound Name	PubChem ID	Class of the compound	IC_{50} (nM)
1	Pralsetinib	129073603	Not Classified	0.4
2	LOXO-292	134823904	Not Classified	1
3	Luminespib	135539077	Benzenoids	1.34
4	Sitravatinib	25212148	Not Classified	1.5
5	Lenvatinib	9823820	Quinolines	1.5
6	RXDX-105	56846693	Not Classified	3
7	Vandetanib	3081361	Diazanaphthalenes	4
8	Alectinib	49806720	Indoles	4.8
9	Crizotinib	11626560	Pyridines	5
10	Sunitinib	5329102	Indoles	6.66
11	Sorafenib	216239	Organooxygen compounds	7.33
12	Regorafenib	11167602	Organooxygen compounds	8.82
13	AD80	71578106	Pyrazoles	9
14	Cabozantinib	25102847	Organooxygen compounds	11
15	Apatinib	11315474	Not Classified	13
16	Ponatinib	24826799	Benzenoids	25.8
17	Nintedanib	135423438	Indoles	34
18	Dovitinib	135398510	Diazinanes	200

Table S2: Calculated global descriptors of hybrid molecules using LACV3P++** energy level.

S. No.	Control and Linked Fragments	Ionization Potential (IP)	Electron Affinity (EA)	Hardness (η)	Softness (S)	Chemical Potential (χ)
1	Pralsetinib	8.320	0.876	3.720	0.268	4.590
2	LF1	8.022	-0.092	4.060	0.246	3.970
3	LF2	8.954	0.800	4.080	0.245	4.880
4	LF21	6.057	0.447	3.108	0.321	2.950
5	LF27	6.067	-0.160	2.630	0.380	3.437
6	LF88	8.368	0.807	3.960	0.252	4.410

Table S3: Hydrogen bond interaction involved between the ligand molecules and the receptor.

S. No.	Control and Linked Fragment	Interacting Residues	Interaction Distance (Å)
1	Pralsetinib	ALA807 … N	1.97
		NH … ALA807	2.55
		SER811 … N	2.21
2	LF1	OH … ALA807	2.23
3	LF2	NH … ALA807	2.06
		ALA807 … O	2.05
4	LF88	N … ALA807	3.83
		NH … ALA807	2.70
		NH … ASN879	2.47

Table S4: In silico activity prediction of control and hybrid molecules against LC-2/ad cell line.

S. No.	Control and linked fragments	IC_{50} (log µM)	IC_{50} (µM)
1	Pralsetinib	3.294	26.950
2	LF1	3.573	35.623
3	LF2	2.082	8.020
4	LF88	1.521	4.576

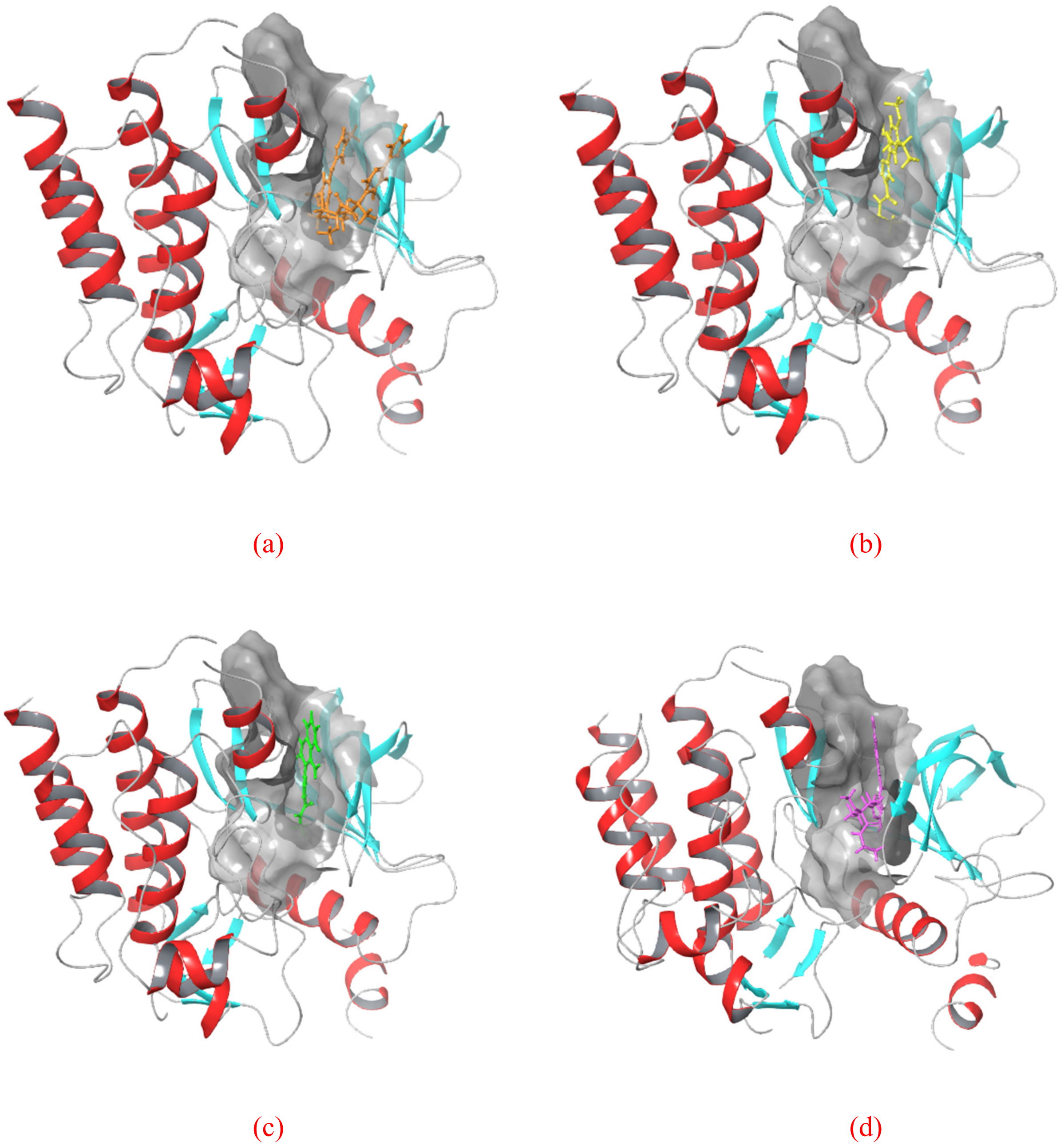

Figure S1: 3D visualization of bound protein ligand complexes: (a) RET-pralsetinib, (b) RET-LF1, (c) RET-LF2 and (d) RET-LF88 complex.

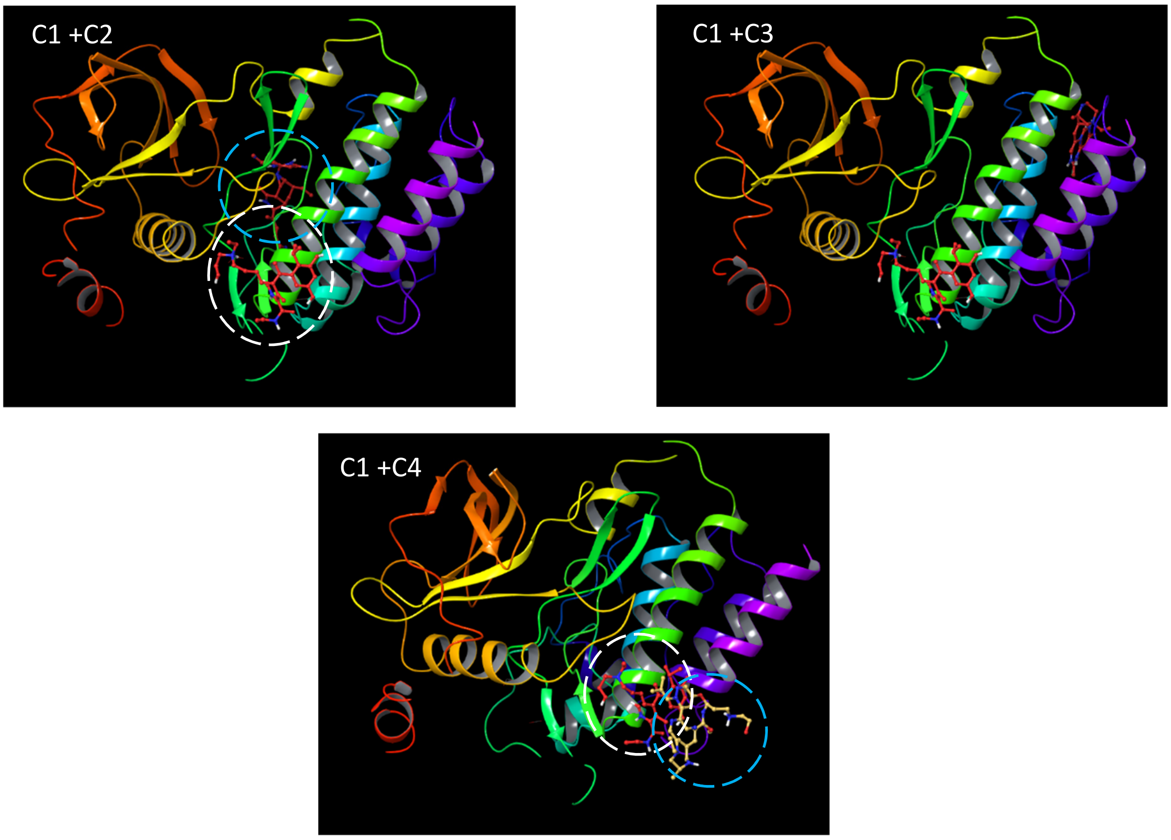

(c)

Figure S2: The synergistic docking of parental compounds where C1, C2, C3 and C4 denotes Luminespib, Pralsetinib, Dovitinib and LOXO-292 respectively. White circle indicates the binding position of compound 1 and the blue circle represents the respective binding mode of the 2nd compound.
